# Neuroprotective Effects of N-Acetylcysteine-Amide (AD4) in a Survival Mouse Model of Paraoxon Intoxication: Targeting Oxidative Stress, Neuroinflammation and Memory Impairments

**DOI:** 10.3390/antiox14121463

**Published:** 2025-12-06

**Authors:** Edurne Urquizu, Marine Cuiller, Georgia Papadopoulou, David Pubill, Demetrio Raldúa, Jordi Camarasa, Elena Escubedo, Raul López-Arnau

**Affiliations:** 1 Department of Pharmacology, Toxicology and Therapeutic Chemistry, Pharmacology Section and Institute of Biomedicine (IBUB), Faculty of Pharmacy, University of Barcelona, 08028 Barcelona, Spain; edurneurquizullop@ub.edu (E.U.); d.pubill@ub.edu (D.P.); jcamarasa@ub.edu (J.C.); eescubedo@ub.edu (E.E.); 2 Institute for Environmental Assessment and Water Research (IDAEA-CSIC), 08034 Barcelona, Spain; drpqam@cid.csic.es

**Keywords:** AD4, paraoxon, memory, oxidative stress, neuroinflammation, organophosphorus

## Abstract

Neurotoxicity induced by organophosphorus (OP) compounds such as paraoxon (POX) leads to severe brain damage and cognitive impairments. Although current treatments alleviate acute cholinergic symptoms, they fail to address secondary neurotoxicity. This study investigated the therapeutic potential of N-acetylcysteine-amide (AD4), a blood–brain-barrier permeable antioxidant, in a survival mouse model of acute POX intoxication. Male Swiss CD-1 mice received POX (4 mg/kg) followed by standard emergency therapy (atropine, pralidoxime and diazepam). AD4 (150 mg/kg) was administered 2 and 6 h post-exposure. AD4 treatment effectively prevented oxidative stress by reducing lipid peroxidation and restoring the expression in hippocampus (HP) and/or prefrontal cortex (PFC) of key antioxidant enzymes such as glutathione peroxidase-1 (GPx-1) and catalase (CAT) suppressed by POX acute exposure. Moreover, AD4 attenuated neuroinflammation in specific hippocampal subregions, as evidenced by reduced Glial Fibrillary Acidic Protein (GFAP) and Ionized Calcium Binding Adaptor Molecule 1 (Iba-1) immunoreactivity. Importantly, AD4 also rescued recognition memory deficits, as assessed by the Novel Object Recognition Test (NORT). In summary, these findings demonstrate that AD4 mitigates oxidative stress, neuroinflammation, and cognitive dysfunction following acute POX intoxication, supporting its potential as an adjuvant therapy for mitigating the secondary neurotoxicity derived from organophosphorus poisoning.

## 1. Introduction

Organophosphorus (OP) compounds are a diverse class of chemical organic compounds that are employed in industry and in agriculture as pesticides and herbicides, being the most normally used insecticides worldwide. Moreover, they have also been used as nerve agents in chemical warfare [1], being employed for the first time in a war context in the 1980s and in terrorist attacks in the 1990s. Several reviews estimated 3 million of intoxications per year with 220,000 deaths, being in the majority intentional, either resulting from a suicidal form or in terrorist attacks [2,3,4], highlighting acute OP poisoning as a significant public health concern.

Parathion is a commonly used and highly toxic OP pesticide that once within the organism, is bioactivated in the liver to its toxic metabolite, paraoxon (POX), which exerts its effects, as other OP compounds, by irreversible inhibiting the enzyme acetylcholinesterase (AChE) [5] through phosphorylation of the serine residue in the active site of the enzyme, thus forming a covalent bond between AChE and the OP compound. This irreversible inhibition leads to excessive acetylcholine (ACh) accumulation in the synaptic cleft, causing cholinergic hyperstimulation and a range of cholinergic symptoms (primary toxicity) [6,7], such as vomiting, diarrhea, miosis, bradycardia, bronchorrhea and focal seizures. Furthermore, high and acute OP exposure may lead to respiratory failure and ultimately the death of the organism [8,9].

The standard emergency treatment for severe acute OP poisoning, including POX, typically involves atropine, which blocks overstimulation of muscarinic receptors caused by excessive ACh accumulation; oximes, which help restore AChE activity through cleaving the covalent bond with the OP compound; and benzodiazepines, to manage and control seizures, enhancing GABAergic signaling [10,11,12]. This treatment primarily addresses acute immediate symptoms, thus increasing the survival rate of the individual. However, exposure to POX and similar OP compounds can trigger secondary neurotoxic effects. As previously discussed, excessive ACh accumulation caused by OP-mediated inhibition of AChE overstimulates muscarinic receptors, initiating focal seizures [13]. These seizures promote excessive glutamate release, leading to NMDA receptor activation and intracellular calcium overload [14,15]. If such hyperactivity persists, status epilepticus (SE) may ensue, resulting in excitotoxic neuronal injury [16,17,18]. Collectively, these processes contribute to oxidative stress, neuroinflammation, memory deficits, and neurodegeneration [5,19,20,21,22]. Importantly, similar long-term neurological outcomes have been documented in humans, including civilians exposed during the Tokyo subway sarin attack (1995) and agricultural workers chronically exposed to OP pesticides, where persistent cognitive deficits have been observed [23,24,25,26]. Unfortunately, current emergency treatments fail to address this so-called secondary neurotoxicity sequalae’s, highlighting the pressing need for new therapeutic strategies. Therefore, the use of survival animal models of OP poisoning is essential for elucidating the mechanisms underlying these long-term effects and for evaluating effective and translational countermeasures.

The brain is highly vulnerable to oxidative stress due to its high metabolic rate, oxygen consumption and its abundance in polyunsaturated fatty acids that can be oxidized [27,28]. Particularly, the hippocampus (HP) and prefrontal cortex (PFC) appear to be especially susceptible to OP compounds, probably due to their high density of cholinergic innervation [29]. In this regard, the oxidative stress induced by OP compounds in both brain areas is thought to contribute to the pathological mechanism of OP poisoning, including neuroinflammation and memory impairments [19,30,31,32]. Therefore, antioxidants such as N-acetylcysteine (NAC) have been used to counteract oxidative stress caused by OP poisonings [33,34]. NAC is a synthetic derivative of the amino acid L-cysteine and serves as a crucial precursor of glutathione (GSH). However, despite presenting a strong therapeutic effect against oxidative stress and inflammation [35,36,37], its ability to cross the blood–brain barrier (BBB) is limited [38,39]. In this sense, scientists engineered a modified form of NAC known as N-acetylcysteine amide (NAC-amide or AD4/NACA) to address this drawback. By altering its structure with a C-α-amidation to increase lipophilicity, AD4 achieves improved ability to cross the BBB [40,41]. Once inside the cells, AD4 has been shown to restore the GSH pool in thiol-depleted cells, potentially due to its copper (Cu^2+^) chelation properties, thereby acting as a potent antioxidant molecule [42]. In fact, AD4 can protect against glutamate cytotoxicity by inhibiting lipid peroxidation and scavenging reactive oxygen species (ROS) [43]. Moreover, AD4 presents anti-inflammatory properties which have been demonstrated in vitro and in vivo [41], through the inhibition of the activation of the MAPK apoptotic pathway and through the regulation of the nuclear translocation of NF-kB [41,44]. Altogether, these results indicate that AD4 has a strong potential as a therapeutic candidate for mitigating OP-induced secondary neurotoxicity.

Therefore, the aim of the present study was to evaluate the antioxidant properties of AD4 (NAC-amide) on glutathione peroxidase 1 (GPx1), catalase (CAT), and 4-hydroxynonenal (4-HNE) protein levels and its anti-inflammatory effects (astroglial and microglial responses) in HP and/or PFC, as well as its therapeutic potential in preventing recognition memory deficits, using a survival mouse model of severe acute POX poisoning.

## 2. Materials and Methods

### 2.1. Animals

Male Swiss CD-1 mice (7–8 weeks old [Janvier, Le Genest-Saint-Isle, France]), weighing 30–40 g, were utilized for the study. The animals were housed in groups of 6–7 per cage under standardized conditions, including a controlled temperature of 22 ± 1 °C and a 12 h light/dark cycle, with unrestricted access to standard food and water. The experimental groups were assigned using block randomization, and the researchers were blinded to group allocation, outcome assessment and data analysis.

To minimize potential confounding factors, animals were randomly assigned to experimental groups and maintained under consistent handling and testing conditions. All measurements were conducted at the same time of the day to avoid circadian influences. Cage positions were kept constant throughout the experiment.

All animal care and experimental procedures adhered to the European Community Council Directive (2010/62/EU) and received approval from the Animal Ethics Committee of the University of Barcelona, under the oversight of the Autonomous Government of Catalonia. This study complies with the ARRIVE guidelines for reporting animal research.

### 2.2. Drugs and Materials

Paraoxon-ethyl was obtained from Cymit quimica (Barcelona, Spain) and dissolved in cold 0.01 M Phosphate-Buffered Saline (PBS). Atropine sulfate and pralidoxime (2-PAM; Pyridine-2-aldoxime methchloride) were purchased from Sigma Aldrich (St. Louis, MO, USA) and prepared in 0.9% NaCl. Diazepam was also sourced from Sigma Aldrich (St. Louis, MO, USA) and dissolved in saline containing 2% DMSO (Scharlab, Barcelona, Spain) and 5% Kolliphor (Sigma Aldrich, St. Louis, MO, USA). All reagents were prepared immediately prior to administration. The protease inhibitor cocktail and phosphatase inhibitor sodium orthovanadate were both acquired from Sigma Aldrich (St. Louis, MO, USA). The novel peptide N-acetylcysteine-amide was generously provided by Prof. Daphne Atlas, at the Hebrew University of Jerusalem (Israel). All other reagents were of analytical grade and obtained from various commercial suppliers.

### 2.3. In Vivo Treatment Schedule—Survival Mouse Model of Acute POX Poisoning

A previously described survival mouse model of POX acute poisoning was employed [19]. Briefly, mice received an acute dose of POX (4 mg/kg) subcutaneously (s.c.), followed 1 min later by an intraperitoneal (i.p.) injection of atropine (4 mg/kg) and 2-PAM (25 mg/kg) to enhance survival. Status epilepticus (S.E.) was observed within 3–5 min of POX administration and monitored for 1 h using a modified Racine Scale (scores 0–6) as previously described [45,46]. To control and terminate seizures, a second i.p. injection of 2-PAM (25 mg/kg) and diazepam (5 mg/kg) was administered 1 h after POX exposure. Furthermore, another group of mice were also administered AD4 (150 mg/kg) i.p. 2 and 6 h after POX treatment. Dosing (150 mg/kg) and the treatment schedule were chosen according to previous effective studies [41,42,47], with minor modifications, as well as available pharmacokinetic data regarding its elimination half-life [48], and to reflect a clinically plausible post-exposure treatment window in an acute high POX exposure scenario (e.g., terrorist attack). The selected time points for behavioral testing, tissue collection, and histological analyses were chosen based on our previously validated mouse model [19] and on literature reports of the temporal progression of organophosphate-induced oxidative stress and neuroinflammation [5,49,50]. To address potential dehydration due to the cholinergic crisis, all mice were given a s.c. injection of 0.9% NaCl at the end of treatment (5 mL/kg). Control group of animals underwent the same protocol but received vehicle injections instead of active compounds. Daily monitoring of weight, appearance, and behavior was conducted for all subjects until euthanasia. A mortality rate of 96% was observed for POX exposed mice, which is in accordance with previous studies using the same treatment protocol [19]. The total sample sizes, along with the rationale for any excluded samples, are detailed in Tables S1–S3 in the Supplementary Material.

### 2.4. Bio- and Neurochemical Assays

#### 2.4.1. Tissue Sample Preparation and Protein Extraction

Seventy-two hours after POX treatment, mice were euthanized via cervical dislocation to collect tissues for protein analysis using Western blotting. The HP and PFC were rapidly dissected out and stored at −80 °C until further processing. Homogenization of tissue samples was performed at 4 °C in 20 volumes of Tris-HCl lysis buffer containing protease and phosphatase inhibitors. The homogenates were vortexed for 15 s and incubated on an orbital shaker at 4 °C for 2 h. Following homogenization, samples were centrifuged at 15,000× *g* at 4 °C for 30 min, and the resulting supernatants were collected and stored at −80 °C. Protein concentrations were determined using the Bio-Rad Protein Reagent (Bio-Rad, Hercules, CA, USA).

#### 2.4.2. Western Blotting and Immunodetection

A standard Western blotting and immunodetection procedure was employed to assess the expression of the antioxidant enzymes GPx1 and CAT. For each sample, 15 μg of protein was mixed with sample buffer containing 0.5 M Tris-HCl (pH 6.8), 10% glycerol, 2% (*w*/*v*) SDS, 5% (*v*/*v*) β-mercaptoethanol, and 0.05% bromophenol blue. The samples were then boiled at 95 °C for 5 min and loaded onto 10% (CAT) or 12% (GPx1) acrylamide gels. Following electrophoresis, proteins were transferred onto polyvinylidene fluoride (PVDF) membranes (Immobilon-P; Merck, Darmstadt, Germany). Membranes were blocked for 1 h at room temperature using WestVision Block and Diluent for Western blots (Vector Laboratories, Newark, NJ, USA), then incubated overnight at 4 °C with the following primary antibodies: rabbit monoclonal anti-GPx1 (ab108427, Abcam, Cambridge, UK; dil. 1:1000) and rabbit monoclonal anti-CAT (#14097, Cell Signaling, Danvers, MA, USA; dil. 1:1000). Membranes were then washed with Tris-buffered saline containing 0.1% Tween-20 and incubated 1 h at room temperature with the horseradish peroxidase-conjugated secondary antibody goat anti-rabbit IgG (ab6721, Abcam, Cambridge, UK; dil. 1:10,000). Immunoreactive proteins were detected using a chemiluminescence-based detection kit (Immobilon Western, Millipore, Burlington, MA, USA) and visualized using a Bio-Rad ChemiDoc XRS gel documentation system (Bio-Rad, Hercules, CA, USA) in accordance with the manufacturer’s instructions. Band densities were quantified with Bio-Rad Image Lab Software (Version 6.1) and normalized to control samples. Protein expression was further normalized using the loading control monoclonal anti-β-actin (A5441, Merck, Darmstadt, Germany; dil. 1:2500). Due to the number of samples, proteins were run on two independent membranes, each including identical control samples and one treated group. The inclusion of the same control samples on both membranes enabled normalization between blots. Results were expressed as a percentage relative to control conditions.

#### 2.4.3. Lipid Peroxidation (4-HNE) ELISA Kit

Mouse HP and PFC were sonicated (0.95 cycle, 95–100 amplitude, 20–25 s) in Tris-HCl lysis buffer plus protease and phosphatase inhibitors. The resulting homogenates were vortexed for 15 s and centrifuged for 15 min at 15,000× *g* at 4 °C. Protein concentration was determined using the Bio-Rad Protein Reagent and 1 mg of protein was used in each 96-well for 4-HNE concentration assay. Quantification of 4-HNE adducts was performed using a competitive ELISA kit (ab238538, Abcam, Cambridge, UK) following the manufacturer’s instructions. Absorbance at 450 nm was recorded using a Benchmark Plus microplate reader (Bio-Rad, Hercules, CA, USA) and data were processed using Microplate Manager Software, version 5.2.1 (Bio-Rad, Hercules, CA, USA). The values were calculated based on the 4-HNE-BSA standard curves and normalized to protein concentration.

#### 2.4.4. Tissue Preparation and Immunohistochemistry

Mice were deeply anesthetized with an i.p. injection of sodium pentobarbital (Dolethal^®^; 150 mg/kg) diluted in PBS 0.01M. The surgical depth of anesthesia was verified by the lack of reflexive response to tail or toe pinches, after which intracardiac perfusion was performed using 4% paraformaldehyde (PFA) prepared in 0.1 M phosphate buffer (PB). Following perfusion, brains were removed and postfixed overnight (O/N) in 4% PFA. On the subsequent days, the storage solution was exchanged by a sucrose gradient, up to 30% sucrose. Coronal sections of 20 μm thickness were collected on a cryostat (Leica Microsystems, Wetzlar, Germany) and stored at −20 °C in cryoprotective solution until further use.

For free-floating immunohistochemistry, sections of interest (HP) were selected and washed 3 times with PBS, followed by 5 washes of 0.5% Triton X-100 in PBS and blocked 1 to 2 h at room temperature in a blocking solution containing 0.2% gelatin, 10% fetal bovine serum (FBS) and 1% Triton X-100 in PBS. After, sections were washed and incubated O/N at 4 °C with the primary antibodies: polyclonal rabbit anti- Glial Fibrilary Acidic Protein (GFAP) (Z0334, Agilent Dako, Santa Clara, CA, USA; dil. 1:1000); polyclonal rabbit anti- Ionized calcium-binding adapter molecule 1 (Iba1) (019-19741, FUJIFILM Wako Chemicals, Richmond, VA, USA; dil. 1:500). After washing, tissue sections were incubated with Alexa-Fluor 568 goat anti-rabbit IgG (Life Technologies, Carlsbad, CA, USA; dil. 1:200) secondary antibody for 1 h at room temperature. Slices were washed afterwards and incubated for 5 min with DAPI (sc-3598, Santa Cruz Biotechnology, Dallas TX, USA; 1.5 μg/mL) for nuclei staining. Slices were finally washed and mounted on Superfrost Plus Adhesion microscope slides (Epredia, Portsmouth, NH, USA) with Fluoromount-G medium (Invitrogen, Carlsbad, CA, USA). For each animal, 4–7 coronal hippocampal sections were selected and imaged using a fluorescence microscope (BX41, Olympus, Hamburg, Germany) with 10× or 20× objectives under identical settings. From each section, three non-overlapping fields per subregion (DG, CA1, CA3) were acquired. Images were analyzed in ImageJ software, version 1.54r (National Institute of Health, Bethesda, MD, USA), converted to 8-bit, and fluorescence intensity was quantified with uniform thresholding. For each animal, mean fluorescence per region was calculated by averaging all fields and sections; these values were then averaged across animals in each group, with all within-animal images treated as technical replicates.

### 2.5. Behavioral Assays

#### 2.5.1. Novel Object Recognition Test (NORT)

NORT was conducted in a V-shaped grey plexiglass maze measuring 37 × 20 × 8 cm, placed in a behavioral testing room with regulated light conditions (30–50 lux). The procedure consisted of 3 sessions, each separated by 24 h, performed on days 9, 10, and 11 after treatment, to evaluate long-term recognition memory. During the first session, mice were habituated by being allowed to freely explore the maze for 9 min. On the second session, 2 objects exactly alike were positioned at the maze’s ends reclined on the walls, and mice were allowed to explore them for 9 min for familiarization, whilst being video monitored. On the third day, one of the objects was replaced with a novel one, and the subjects were given another 9 min exploration period in the maze under video recording. The design of the objects varied in texture, shape and color, and was validated beforehand to ensure no intrinsic preference. The maze and objects were cleaned with 70% ethanol between subjects to remove any residual odors. Preference was assessed using the discrimination index (DI), calculated as the difference between the exploration times of the novel and familiar objects divided by the total exploration time. A lower DI reflected reduced exploration time of the novel object, indicating an impairment of the recognition memory.

#### 2.5.2. Horizontal Locomotor Activity (HLA)

Four days after treatment (Day 5), HLA was performed to measure locomotor activity. Mice were introduced into a black Plexiglas arena (25 × 25 × 40 cm) within a dedicated behavioral testing room. The environment was maintained with continuous white noise and reduced light intensity to minimize external disruptions. Basal locomotor activity was monitored for 1 h using a digital camera system and a movement-tracking software program (Smart v3.0, Panlab, Cornellá, Spain). Locomotor activity was quantified by analyzing the total distance travelled by each subject.

### 2.6. Data Statistical Analysis

All results are presented as mean ± standard error of the mean (S.E.M.). A normality test was conducted prior to analysis, focusing on the Shapiro–Wilk test due to its suitability for small sample sizes. For datasets following normal and parametric distribution, statistical differences were evaluated using one-way ANOVA, followed by Tuckey’s post hoc test for multiple comparisons. When data were not normally distributed, the Kruskal–Wallis test followed by Dunn’s post hoc test was applied. The significance threshold (α) was set at 0.05. The statistical analyses were performed with GraphPad Prism (version 8.0) software.

Animals were included in the study if they met the established criteria of healthy status, appropriate age and body weight within the target range at the beginning of the experiment. Animals that died during the treatment period were excluded from subsequent analyses. In immunohistochemistry experiments, samples were excluded if samples were damaged or unsuitable for proper evaluation. These exclusion criteria were defined a priori.

Outliers were identified and excluded using the ROUT method with a Q value of 0.1%. Apart from these predefined exclusions, no additional animals or data points were omitted.

Sample sizes differed across experimental approaches due to the inherent variability and technical constraints of each method. Behavioral experiments employed group sizes derived from an a priori power analysis (G*Power 3.1.9.4; α = 0.05, power = 0.80, effect size f = 0.40), resulting in N values comparable to those used in the present study. Biochemical analyses (ELISA, Western blot) were performed with 6–7 animals per group, a range consistent with previous OP-related studies [19,35,51,52] and appropriate for assays with lower technical variability and f > 0.40–0.50. Immunohistochemical analyses showed some variation in N due to occasional tissue loss or justified exclusion of outliers; however, each animal contributed multiple averaged technical replicates, ensuring robustness while respecting ethical standards.

## 3. Results

### 3.1. AD4 Reverses the POX-Induced Reduction in GPx1 Expression and Prevents the Increase in Hippocampal 4-HNE Levels

**Figure 1:**
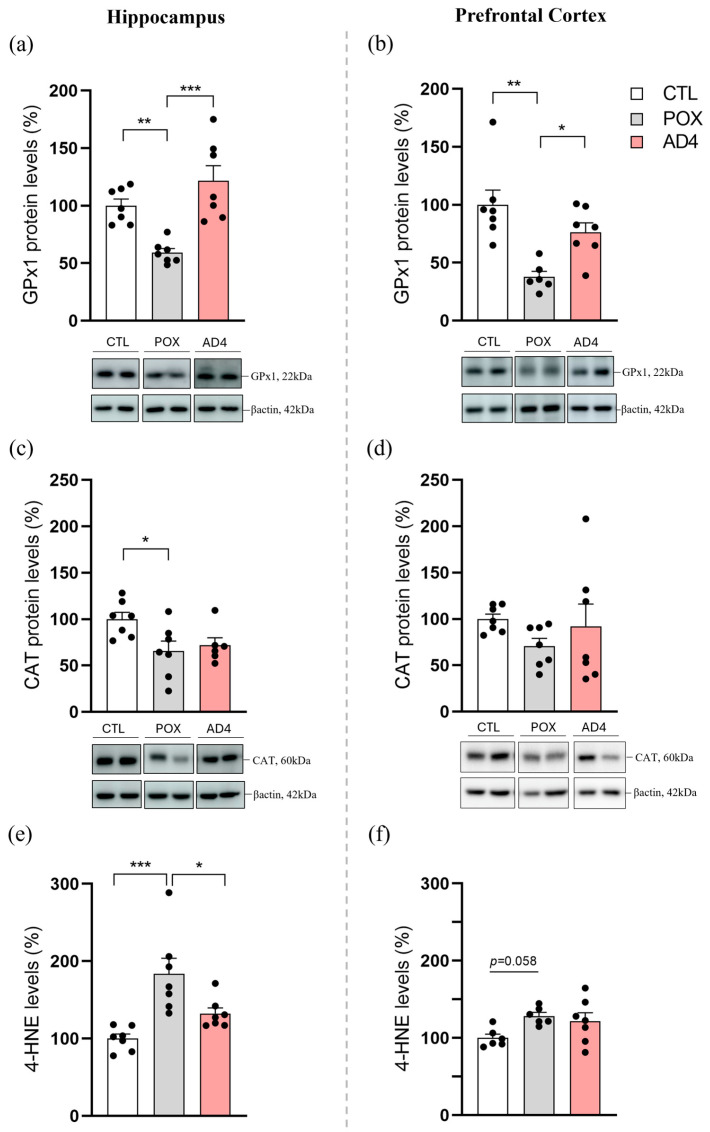
Percentage levels of oxidative stress-related enzymes (**a**–**d**) and oxidative stress markers (**e**,**f**) in the hippocampus (HP) and prefrontal cortex (PFC) of control (CTL), paraoxon-exposed (POX; 4 mg/kg) and AD4-treated (AD4; 150 mg/kg 2 h and 6 h post-POX) groups. Changes in protein expression of glutathione peroxidase 1 (GPx1), catalase (CAT) and 4-hydroxynonenal (4-HNE) were assessed 72 h post-treatment. Values are shown as mean ± standard error of the mean (SEM). Statistical significance is indicated as * *p* < 0.05, ** *p* < 0.01, *** *p* < 0.001. Group sizes were N = 6–7. Representative blot images are displayed beneath each graph (**a**–**d**).

In order to test the capability of AD4-treatment (150 mg/kg, 2 h and 6 h post-POX) to partial or totally counteract the oxidative stress caused by acute POX intoxication (4 mg/kg), we analyzed the protein expression of two important antioxidant enzymes in HP and PFC 72 h after treatment. As observed in Figure 1a, one-way ANOVA revealed a significant effect of treatment on the protein expression of the antioxidant enzyme GPx1 in HP (F_(2, 18)_ = 3.840; *p* < 0.05). Specifically, acute POX administration resulted in a significant decrease in the protein levels in HP (*p* < 0.01), an effect that was completely reversed by treatment with the AD4 peptide (*p* < 0.001). Moreover, despite the significant reduction in CAT protein expression observed in HP following intoxication with POX (F_(2, 17)_ = 4.325; *p* < 0.05), the subsequent administration of AD4 failed to significantly reverse this effect (Figure 1c). Regarding the expression of the enzyme GPx1 in PFC, the Kruskal–Wallis test revealed a statistically significant difference among the three treatment groups (H(2) = 11.78, *p* < 0.05). Acute POX administration significantly reduced GPx1 expression levels (*p* < 0.01), whereas AD4 treatment prevented this decrease (*p* < 0.05; Figure 1b). However, neither POX exposure nor AD4 treatment could significantly affect CAT expression in the PFC (F_(2, 18)_ = 1.017; *p* > 0.05; Figure 1d).

Furthermore, the lipid peroxidation marker 4-HNE was assessed through an ELISA kit. As expected, a significant increase in 4-HNE protein adducts in HP (*p* < 0.001) was observed after POX treatment. Importantly, the administration of the novel peptide AD4 after POX intoxication prevented this increase (*p* < 0.05), thus protecting against OP induced oxidative stress (F_(2, 18)_ = 10.95; *p* < 0.001; Figure 1e). Nevertheless, despite observing a similar tendency in PFC, the effect of treatment was not statistically significant (F_(2, 16)_ = 3.455; *p* = 0.0566; Figure 1f).

### 3.2. AD4 Mitigates POX-Induced Astroglial and Microglial Responses in the Hippocampus

**Figure 2:**
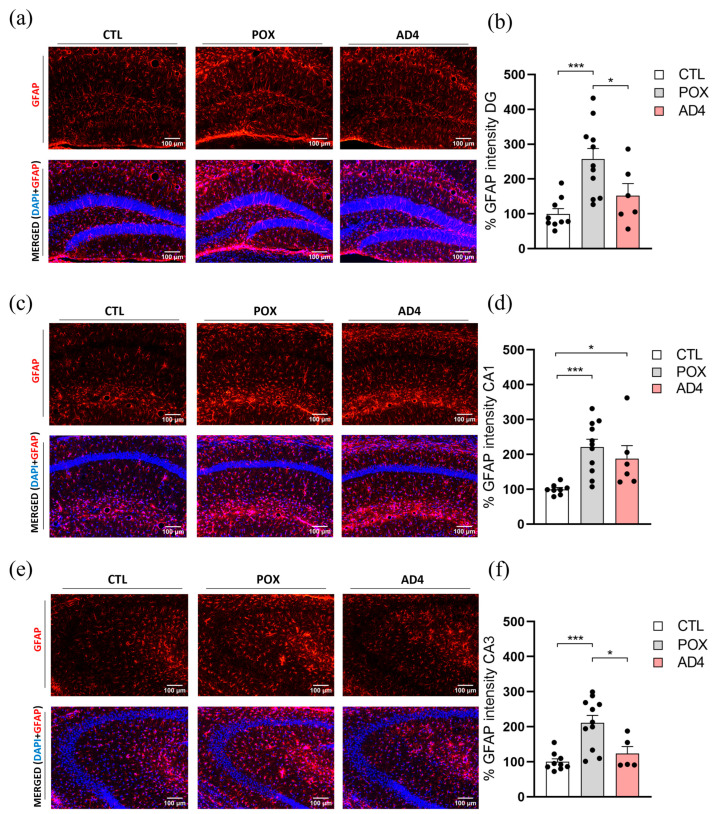
Neuroinflammatory response evaluation 72 h after treatment. Representative images of the immunohistochemical detection of the astrocytic marker Glial Fibrillary Acidic Protein (GFAP) in combination with DAPI for nuclear detection. Figures show the hippocampal regions of Dentate Gyrus (DG) (**a**), CA1 (**c**) and CA3 (**e**) of CTL, POX and AD4-treatedAD4 mice. Graphic representations of fluorescence quantification are shown in figures (**b**,**d**,**f**). Data are expressed as mean ± SEM, N = 5–11/group, * *p* < 0.05, *** *p* < 0.001.

To explore the potential therapeutic effect of AD4 administration on the inflammatory impact of POX intoxication, we measured GFAP and Iba-1 intensity in HP, the region most affected by POX and most responsive to AD4 treatment in terms of lipid peroxidation. One-way ANOVA revealed that GFAP, a key protein found in astrocytic cells and a well-established indicator of astrogliosis, was significantly increased in DG (F_(2, 23)_ = 9.436, *p* < 0.01) and CA3 (F_(2, 22)_ = 11.56, *p* < 0.001). In the CA1 region, where data did not meet the assumptions of normality, a Kruskal–Wallis test was performed instead, revealing a significant group effect (H(2) = 14.07, *p* < 0.001). Together, these results indicate that GFAP levels were elevated across the three principal hippocampal subregions. Moreover, Tuckey’s post hoc test revealed that AD4-treatment was able to significantly reduce the inflammation caused by POX poisoning in DG and CA3 areas (Figure 2a,c), but not in CA1 (Figure 2b).

**Figure 3:**
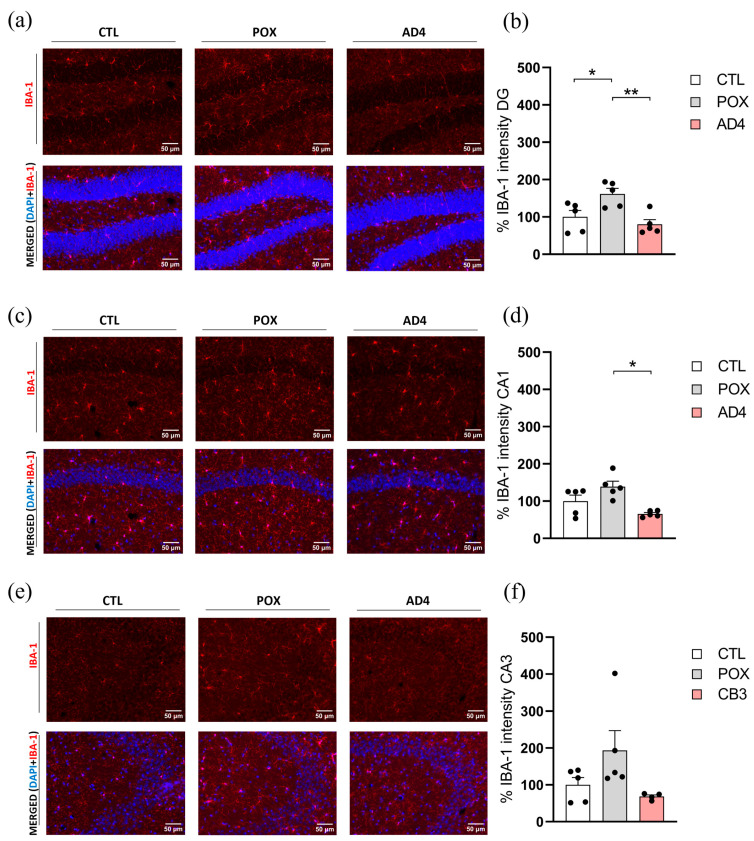
Evaluation of neuroinflammation though the microglial marker Ionized calcium-binding adapter molecule 1 (Iba-1) 72 h post-treatment. Figures (**a**,**c**,**e**) are representative images of the immunofluorescence staining assessed in each treatment group in the regions of DG, CA1 and CA3 of the HP, respectively. Quantification of the fluorescence intensity is represented through the graphics appearing in figures (**b**,**d**,**f**). Results are shown as mean ± SEM, N = 4–5/group, * *p* < 0.05, ** *p* < 0.01.

On the other hand, one-way ANOVA also revealed a significant effect of the treatment in Iba-1 expression, a microglial marker, in the DG (F_(2,12)_ = 7.991, *p* < 0.01). Furthermore, Tukey’s post hoc analysis showed that AD4 administration significantly reduced POX-induced inflammation in the DG (Figure 3a). However, this effect was not observed neither in CA1 nor CA3 hippocampal areas (Figure 3b,c).

### 3.3. AD4 Improves Recognition Memory Deficits in POX-Exposed Surviving Mice

**Figure 4:**
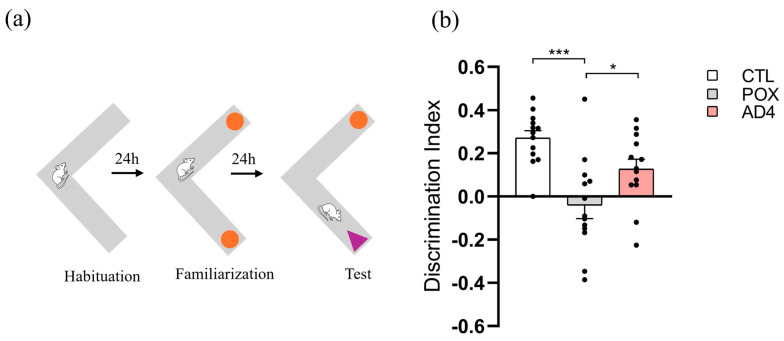
Assessment of long-term recognition memory by Novel Object Recognition Test (NORT) after POX and AD4 treatment. Discrimination Index (DI) represents the rate of discrimination between the familiar (orange circles) and novel object (purple triangle). Panel (**a**) schematically shows the three phases of the test and panel (**b**) graphically represents the DI calculated from every group. Bars present the mean ± SEM, Tukey’s multiple-comparisons test; * *p* < 0.05, *** *p* < 0.001 vs. the control group, N = 13/group.

During the NORT test session (Figure 4a), one-way ANOVA indicated no significant differences between the three groups in the total exploration time of both objects (F_(2, 36)_ = 1.357, *p* > 0.05; Figure S1). However, the analysis revealed a statistically significant effect of the treatment variable on the DI (F_(2, 36)_ = 12.82, *p* < 0.001). Specifically, as shown in Figure 4b, the DI score was significantly reduced in the POX-treated group compared to controls (*p* < 0.001), but this reduction was partially prevented in the AD4-treated group (*p* < 0.05). These results suggest that treatment with AD4 mitigates, at least partially, the decline in recognition memory induced by acute POX intoxication.

### 3.4. AD4 Does Not Affect Basal Hyperlocomotion Induced by Acute POX Intoxication

As shown in Figure S2A, one-way ANOVA of the total distance travelled yielded a significant effect of the variable Treatment (F_(2, 25)_ = 4.785, *p* < 0.05). Both POX and AD4 groups yielded a significant increase in the distance travelled for 1 h.

Figure S2B represents the HLA time-course profile. Test of two-way ANOVA revealed a significant effect of the variables Time (F_(4.672, 116.8)_ = 72.51, *p* < 0.001) and Treatment (F_(2, 25)_ = 4.785, *p* < 0.05). However, there are no significant differences for the interaction between both factors (F_(22, 275)_ = 0.4846, *p* > 0.05).

### 3.5. Summary Results

As reflected in Table 1, POX exposure produced a clear oxidative and inflammatory response, shown by decreased GPx1, increased 4-HNE levels, and elevated GFAP and Iba1 expression. These changes were accompanied by impaired recognition memory in NORT. AD4 treatment effectively reversed these alterations, restoring antioxidant enzyme protein levels, reducing lipid peroxidation and glial activity, accompanied by a restoration of the memory deficit.

**Table 1:** Summary table of the principal neuroprotective effects of AD4 treatment on POX-survival mouse model. The symbols represent increase (↑), decrease (↓) and non significant changes (ns.).

Effect	Effect of POX				Restored Parameters by AD4			
Oxidative stress	HP		PFC		HP		PFC	
	GPx1 ↓		GPx1 ↓		GPx1		GPx1	
	CAT ↓		CAT (ns.)		-		-	
	4-HNE ↑		4-HNE (ns.)		4-HNE		-	
Neuroinflammation	DG (HP)	CA1 (HP)		CA3 (HP)	DG (HP)	CA1 (HP)		CA3 (HP)
	GFAP ↑	GFAP ↑		GFAP ↑	GFAP	-		GFAP
	IBA-1 ↑	IBA-1 (ns.)		IBA-1(ns.)	IBA-1	-		-
Cognitive deficits	NORT DI ↓				NORT DI			

## 4. Discussion

Previous research has demonstrated that OP poisoning, such as that induced by POX, leads to secondary neurotoxicity that is not fully mitigated by current standard emergency treatments [19,53]. This highlights the critical need for additional therapies capable of addressing the long-term neurological consequences of OP exposure (oxidative stress, neuroinflammation, cognitive impairments, neurodegeneration…). In this context, this study evaluated the therapeutic potential of AD4, a novel antioxidant peptide, as a neuroprotective agent in a survival mouse model of acute POX poisoning.

Since it is well established that OP poisoning induces oxidative stress in mice [34,54], we first aimed to determine if the novel peptide AD4 could counteract this redox imbalance in the mouse brain. As previously described [19], our findings demonstrate that POX intoxication causes a disturbance in the intracellular redox state, altering antioxidant enzymes such as GPx1 and CAT in cortical and/or hippocampal subregions. Particularly, GPx1 exerts direct control over cellular redox homeostasis, both by catalyzing H_2_O_2_ removal and by promoting oxidation of GSH, the predominant low-molecular-weight thiol in mammalian cells [55]. Moreover, GPx1 is a key antioxidant enzyme that, under many physiological conditions, demonstrates greater efficiency than CAT in eliminating intracellular peroxides [55]. Our results demonstrated that AD4 could prevent the decrease in the levels of the antioxidant enzyme GPx1 in HP and PFC, keeping its expression to normal levels, but no effect on CAT imbalance was observed. To our knowledge, this is the first study carried out using AD4 as a complementary therapeutical drug in a survival mouse model of acute OP poisoning. However, data obtained can be supported by other’s research using its precursor, NAC. For instance, John and colleagues (2019) demonstrated that repeated NAC injections (150 mg/kg, i.p. × 21 days) combined with the standard emergency treatment was able to prevent the downregulation of GPx brain levels in mice against sub-acute diisopropyl phosphorofluoridate (DFP) poisoning (21 days of repeated exposure) [35]. When interpreting these findings, it is important to consider the differences in experimental design. Despite the use of an equal antioxidant dose (150 mg/kg, i.p.), our protocol consisted of only two administrations (2 and 6 h after POX exposure) and involved an acute, high-dose of the OP compound, in contrast to the repeated NAC (21 days) and sub-acute DFP exposure described by John and colleagues (2019) [35]. Collectively, these results highlight the improved antioxidant capacity of AD4, a BBB-permeable analog of NAC, under conditions of severe acute POX intoxication.

The increased generation of ROS under oxidative stress can lead to lipid peroxidation due to their high reactivity. Consequently, there is an increased endogenous formation of secondary reactive aldehydes, such as 4-HNE [56]. In this study, high concentrations of this cytotoxic 4-HNE protein adducts in the HP of POX-treated mice confirmed the presence of pronounced lipid peroxidation. Moreover, our findings also provide evidence that AD4 administration significantly reduced 4-HNE levels in POX poisoned mice. These results are consistent with previous studies, in which the use of the antioxidant NAC in front of an acute or chronic OP treatment (DFP, pirimiphos-methyl, fenthion or O-Ethyl S-diisopropylaminomethyl—VX nerve agent) in mice, avoided the increase in lipid peroxidation markers, such as malonaldehyde (MDA) [34,35,57,58]. Taken together, our results clearly demonstrate that the oxidative stress observed in HP of POX surviving mice could be prevented or attenuated with the administration of the antioxidant AD4, which may dampen or even avoid future neurological consequences derived from this state. Although 4-HNE changes in the PFC did not reach statistical significance in the present study (*p* = 0.058), a trend toward increased levels was observed, consistent with previous findings in our laboratory [19]. Differences in statistical approach and methodology may contribute to these discrepancies, and further studies are needed to clarify potential regional effects of AD4.

An additional consideration concerns the interpretation of antioxidant enzyme changes. Although both CAT and GPx1 are recognized targets of OP-induced oxidative stress [5,35,49,59], in the present study CAT was only reduced in the hippocampus and was not modified by AD4, while GPx1 showed clearer vulnerability and partial restoration after treatment in all regions tested. These region- and enzyme-specific differences suggest that the effects observed are unlikely to reflect a uniform loss of neuronal proteins due to neuronal death. Instead, they probably result from distinct susceptibilities to oxidative/nitrosative modification and protein destabilization following POX exposure. Therefore, the improvement in GPx1 levels after AD4 likely reflects a general amelioration of redox imbalance [40,41,44] rather than a selective protective mechanism. However, further studies will be required to determine whether AD4 directly modulates antioxidant enzyme expression or stability, as well as cell death.

Oxidative stress and neuroinflammation following OP exposure are tightly interconnected processes. The oxidative insults activate redox sensitive signaling pathways that are associated with neuronal cell death and inflammation [60,61,62]. In turn, microglial and astrocytic activation release pro-inflammatory cytokines, generating ROS. The reciprocal interaction establishes a cycle where oxidative stress amplifies neuroinflammation and inflammatory mediators further enhance ROS generation, exacerbating neuronal dysfunction and injury [63,64,65]. For instance, it has been consistently reported that OP exposure induces early activation of astrocytes and microglia, as evidenced by upregulated GFAP and Iba-1 expression and increased release of pro-inflammatory cytokines in rodents exposed to sarin, chlorpyrifos, dichlorvos, dimethoate and malathion [64]. Several studies support this finding, showing that an acute dose of OP triggers a neuroinflammatory response in rodents [19,65,66,67]. In agreement with previous studies, our results demonstrate that an acute dose of POX, together with the corresponding emergency treatment, still generates a significant increase in the expression of reactive astrogliosis biomarkers and microglial activation markers in specific hippocampal subregions, suggesting a state of neuroinflammation. In this scenario, various research groups have analyzed the potential of antioxidants to counteract OP-induced neuroinflammation. For instance, natural antioxidants such as thymoquinone and lycopene significantly decreased pro-inflammatory cytokines in rats chronically treated with chlorpyrifos [68], while erdosteine partially restored cytokine balance in diazinon-poisoned rats [69]. Other antioxidant approaches have also shown beneficial effects in mitigating OP toxicity: catalytic antioxidants such as AEOL 10150 attenuated Iba-1 immunoreactivity and reduced hippocampal IL-6 levels after soman exposure [70] and diapocynin effectively decreased astrogliosis and a wide range of cytokines in the HP after poisoning with DFP [71]. The present study demonstrates that AD4 treatment prevents the increase in GFAP immunoreactivity in both the DG and CA3 subregions of the HP, thereby attenuating astrogliosis in key brain regions involved in cognitive processes. In addition, AD4 also prevents Iba-1 overexpression in the hippocampal DG area of POX poisoned mice, indicating a protective effect against the microgliosis induced by POX exposure. Altogether, these results support the potential of AD4 as an antioxidant intervention capable of diminishing POX-induced astro- and microglial activation in HP, thereby mitigating OP-induced neuroinflammation.

Clinical and epidemiological studies involving civilians and/or military personnel exposed to OP compounds, either through acute poisoning or chronic exposure, have shown associations with persistent neurological and neurobehavioral symptoms, including impairments in learning and memory [23,26,72,73,74,75]. Experimental data in rodents after acute exposure to OP compounds are consistent with human clinical data, showing that a single injection of POX [19,53], or DFP [65] can cause memory and learning deficits. Despite the lack of a fully defined role, the HP constitutes a critical region for the formation and consolidation of long-term episodic memory [76]. Therefore, the cognitive deficits observed in the NORT performance of POX-treated mice may be directly associated with the neurotoxic alterations found in this region, including increased oxidative stress and sustained neuroinflammation. As previously discussed, our results demonstrate that AD4 primarily preserves hippocampal integrity by preventing oxidative stress and reducing neuroinflammatory processes in a survival mouse model of POX poisoning. Furthermore, previous studies have provided evidence that AD4 exerts neuroprotective effects, significantly preventing the cognitive decline in mice subjected to mild traumatic brain injury, as assessed by NORT [77]. Consequently, we next evaluated whether AD4 could mitigate the memory impairment observed in POX-surviving mice. Indeed, our results demonstrate that AD4 was able to improve novel object recognition performance in mice previously exposed to POX, suggesting a protective effect against the memory deficits induced by acute and severe POX intoxication.

Although AD4 improved memory and reduced oxidative stress after paraoxon exposure, locomotor hyperactivity remained unchanged. This might reflect limited protection in dopaminergic areas such as the striatum, at the treatment schedule used in the present study. Another possibility is residual cortical hyperexcitability that the current AD4 treatment cannot dampen. Based on this last hypothesis, a combined treatment with NMDA receptor antagonist, such as memantine, could potentially mitigate this effect. In addition, it is known that POX exposure induces dose-dependent motor disturbances such as tremors, hyperactivity, and involuntary movements [78,79]. These motor effects can also persist, as POX-induced neuronal plasticity and changes in nicotinic receptor subunit expression may last for days or longer [79,80]. In this context, the persistence of hyperactivity despite AD4 treatment suggests that, although AD4 reduces oxidative stress and neuroinflammation, it may not substantially influence cholinergic or other motor-related pathways affected by POX. Nevertheless, additional studies might help to clarify this lack of AD4 restoring locomotor effects.

From a translational perspective, it is important to note that AD4 remains at a preclinical stage and does not currently have an established regulatory status or clinical evaluation. As with other experimental neuroprotective strategies, additional studies addressing safety, pharmacokinetics, and feasibility will be necessary before considering potential clinical translation.

## 5. Conclusions

In summary, the reported survival mouse model of acute POX intoxication successfully recapitulates key features previously described in humans and rodents, underscoring its translational value for investigations and therapeutic screening. Importantly, treatment with the antioxidant peptide AD4, a BBB-crossing NAC analog, significantly mitigated oxidative stress and neuroinflammatory responses, which likely underlie the improved cognitive outcomes observed in mice. The present study highlights the capacity of AD4 to mitigate certain aspects of secondary neurotoxicity associated with acute POX poisoning, particularly in selected hippocampal subregions. Taken together, these results support AD4 as a promising therapeutic candidate to counteract OP-induced oxidative damage, neuroinflammation and memory impairments.

## Data Availability

The original data presented in the study are openly available in CORA—*Repositori de Dades de Recerca* at https://dataverse.csuc.cat/dataset.xhtml?persistentId=doi:10.34810/data2785 (accessed on 26 October 2025).

## Supplementary Materials

The following supporting information can be downloaded at https://www.mdpi.com/article/10.3390/antiox14121463/s1, Figure S1: Exploration time in NORT; Figure S2: Basal locomotor activity. Table S1: Sample sizes and exclusion criteria for biochemical analyses (Western blot and ELISA); Table S2: Sample sizes and exclusion criteria for immunohistochemistry analyses (GFAP and IBA-1); Table S3: Sample sizes and exclusion criteria for behavioral analyses.

## Abbreviations

The following abbreviations are used in this manuscript:

2-PAM	Pralidoxime
4-HNE	4-Hydroxynonenal
Ach	Acetylcholine
AChE	Acetylcholinesterase
AD4	N-acetylcysteine-amide
BBB	Blood–brain barrier
CAT	Catalase
DG	Dentate Gyrus
DFP	Diisopropyl phosphorofluoridate
DI	Discrimination Index
FBS	Fetal Bovine Serum
GFAP	Glial Fibrilary Acidic Protein
GSH	Glutathione
GPx1	Glutathione Peroxidase 1
HP	Hippocampus
HLA	Horizontal Locomotor Activity
i.p.	Intraperitoneal
Iba-1	Ionized Calcium Binding Adaptor Molecule 1
MDA	Malonaldehyde
NAC	N-acetylcysteine
NORT	Novel Object Recognition Test
OP	Organophosphorus
O/N	Overnight
PFA	Paraformaldehyde
POX	Paraoxon
PB	Phosphate Buffer
PBS	Phosphate-Buffered Saline
PFC	Prefrontal Cortex
ROS	Reactive Oxygen Species
SEM	Standard Error of the Mean
S.E.	Status Epilepticus
s.c.	Subcutaneously
