# Supplementary material for: Neuroprotective Effects of N-Acetylcysteine-Amide (AD4) in a Survival Mouse Model of Paraoxon Intoxication: Targeting Oxidative Stress, Neuroinflammation and Memory Impairments
Source: Antioxidants (Basel). 2025 Dec 6;14(12):1463. doi: 10.3390/antiox14121463 (PMC12730126; doi:10.3390/antiox14121463)

SUPPLEMENTARY MATERIAL

Neuroprotective Effects of N-Acetylcysteine-Amide (AD4) in a Survival Mouse Model of Paraoxon Intoxication: Targeting Oxidative Stress, Neuroinflammation and Memory Impairments

Edurne Urquizu [1], Marine Cuiller [1], Georgia Papadopoulou [1], David Pubill [1], Demetrio Raldúa [2], Jordi Camarasa [1], Elena Escubedo [1] and Raul López-Arnau [1,*]

[1] *Department of Pharmacology, Toxicology and Therapeutic Chemistry, Pharmacology Section and Institute of Biomedicine (IBUB), Faculty of Pharmacy, University of Barcelona, 08028 Barcelona, Spain; edurneurquizullop@ub.edu (E.U.); d.pubill@ub.edu (D.P.); jcamarasa@ub.edu (J.C.); eescubedo@ub.edu (E.E.)*

[2] *Institute for Environmental Assessment and Water Research (IDAEA-CSIC), 08034 Barcelona, Spain; drpqam@cid.csic.es*

* Correspondence: raullopezarnau@ub.edu

Table S1. Sample sizes and exclusion criteria for biochemical analyses (Western blot and ELISA).

Assay	Region	CTL	POX	AD4	Total N	Exclusion criteria
**WB GPx1**	Hippocampus	7	7	7	21	—
**WB GPx1**	Prefrontal cortex	7	6	7	20	*One POX animal died.*
**WB CAT**	Hippocampus	7	7	6	20	*One AD4 outlier excluded.*
**WB CAT**	Prefrontal cortex	7	7	7	21	—
**ELISA 4-HNE**	Hippocampus	7	7	7	21	—
**ELISA 4-HNE**	Prefrontal cortex	6	6	7	19	*One CTL and One POX excluded – signal loss*

Table S2. Sample sizes and exclusion criteria for immunohistochemistry analyses (GFAP and IBA-1).

Assay	Region	CTL	POX	AD4	Total N	Exclusion criteria
**GFAP**	Hippocampus – DG	9	11	6	26	*Tissue integrity loss*
	Hippocampus – CA1	8	11	6	25	*Tissue integrity loss*
	Hippocampus – CA3	9	11	5	25	*Tissue integrity loss*
**IBA-1**	Hippocampus – DG	5	5	5	15	*One AD4 outlier excluded.*
	Hippocampus – CA1	5	5	5	15	*One AD4 outlier excluded.*
	Hippocampus – CA3	5	5	4	14	*One AD4 outlier excluded.*

Table S3. Sample sizes and exclusion criteria for behavioral analyses.

Test	CTL	POX	AD4	Total N	Exclusion criteria
**HLA**	10	9	9	30	*One POX and one AD4 outlier excluded.*
**NORT**	13	13	13	39	*One CTL outlier excluded; one POX and one AD4 animal died during treatment.*

Figure S1. Exploration time (in seconds) for each experimental group during the test phase of the NOR test. Data are presented as mean ± SEM, with N=13 mice per group.

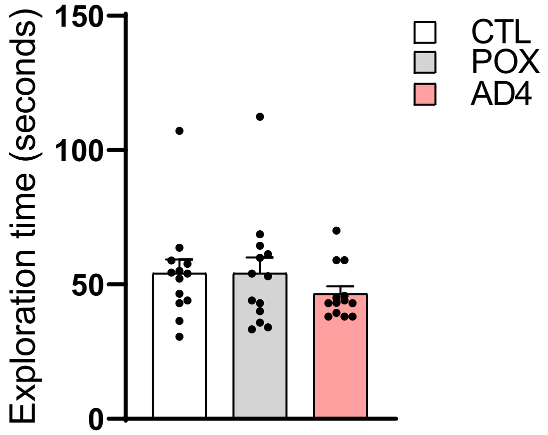

Figure S2. Basal locomotor activity (Day 5) of Swiss CD-1 male mice treated with POX plus standard emergency treatment (POX), mice treated with POX plus emergency treatment and AD4 (AD4), and vehicle-treated mice (CTL). (A) Total distance travelled (cm) for 60 minutes and (B) distance travelled each 5 minutes by each group. Bars represent the mean ± SEM, N=9-10/group; **p*<0.05 vs the control group.

(A) (B)

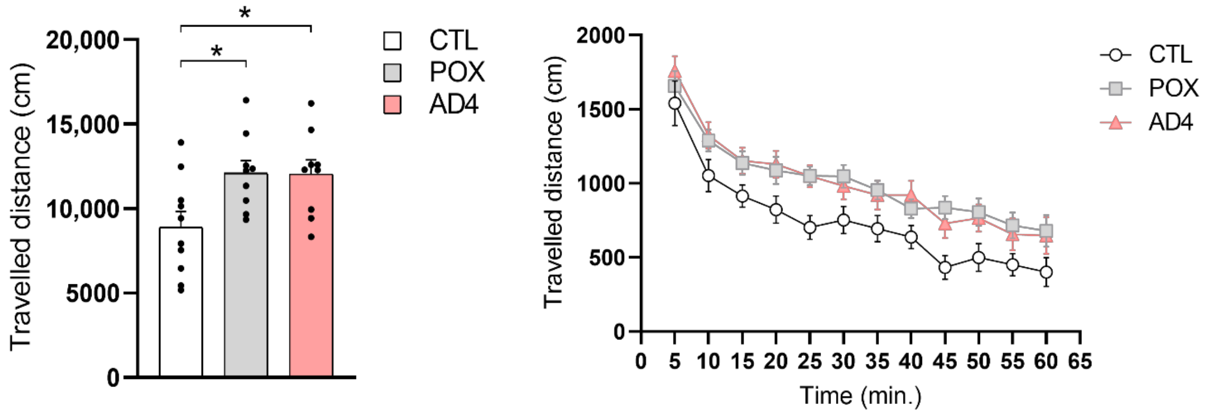

Supplement: Supplementary file 1 [file antioxidants-14-01463-s001.zip › antioxidants-3981343-supplementary.pdf]
